# Efficacy of Oxytetracycline Hydrocortisone-Soaked Gauze Pack on Postoperative Sequelae in Lower Third Molar Surgery: A Prospective Study

**DOI:** 10.7759/cureus.52245

**Published:** 2024-01-14

**Authors:** Sai Krishna, Rajprakash Bhaskaran, Santhosh P Kumar, Murugesan Krishnan

**Affiliations:** 1 Oral and Maxillofacial Surgery, Saveetha Dental College and Hospitals, Saveetha Institute of Medical and Technical Sciences, Saveetha University, Chennai, IND

**Keywords:** impacted teeth, quality of life, trismus, innovative technique, novel dressing, oxytetracycline hydrocortisone soaked gauze, postoperative pain, impacted lower molar, postoperative swelling

## Abstract

Background

Though various advancements came into the field of surgery to do the atraumatic procedure, post-operative pain, and swelling are unavoidable complications. Hence, various medicaments are packed in the extracted third molar sockets to prevent these post-operative complications.

Aim

The study aimed to evaluate the efficacy of oxytetracycline hydrocortisone-soaked gauze in reducing post-operative pain and swelling compared to conventional surgical procedures without any packing in patients undergoing surgical extraction of the impacted mandibular third molars.

Materials and methods

The study was conducted in the Department of Oral and Maxillofacial Surgery at Saveetha Dental College and Hospitals, Chennai. In this study, 50 patients were randomly included in two groups of 25 participants each. In group A, oxytetracycline hydrocortisone-soaked gauze was placed, and in group B, conventional closure was done without any pack after surgical removal of impacted mandibular third molars. Post-operative pain was assessed on days one, three, and five using a 10-point visual analog scale. Post-operative swelling was assessed on the third and seventh days using a four-point swelling measurement. Data analysis was done using SPSS (IBM Corp. Armonk, NY). A p-value less than 0.05 was considered statistically significant. Independent sample t-test was done to compare the outcomes between the two groups.

Results

The results demonstrated that group A (Oxytetracycline Hydrocortisone-soaked gauze) showed superior pain reduction compared to group B (conventional closure) at all post-operative intervals (P=0.001). Moreover, group A exhibited reduced swelling, resulting in higher patient satisfaction levels compared to group B on the third post-operative day (P=0.001).

Conclusion

It can be concluded from the study that there was a significant reduction in post-operative pain and swelling with the use of oxytetracycline hydrocortisone-soaked gauze, as it acts like a local drug delivery system in patients undergoing impacted mandibular third molar surgeries.

## Introduction

Surgical extraction of mandibular molars is a common dental procedure often associated with post-operative pain and discomfort [1]. Swelling, post-operative pain, and trismus are the most common complications that occur after the surgical extraction of lower impacted third molars [2]. Efforts to alleviate pain and enhance wound healing have led to the exploration of various adjunctive therapies, and various intra-socket antibiotics have been tried in extraction sockets [3]. Topical tetracycline has been shown to reduce post-operative complications compared to routine dental extraction [4].

Oxytetracyclines belong to the broad-spectrum group of antibiotics, which usually inhibit protein synthesis and prevent bacterial growth. They primarily function against various gram-positive and gram-negative species as bacteriostatic agents [5]. In gram-negative bacteria, the drug moves through the porin channels, accumulates in the periplasmic space, binds to the 30S ribosomal subunit, and prevents the union of t-RNA with bacterial ribosome [6]. Matrix metalloproteinases are the substances that usually regulate the inflammatory process and lead to inflammation and bone loss. Apart from the antimicrobial properties, tetracyclines will bind to the matrix metalloproteinases and regulate the inflammation. Hence, tetracyclines were used in dermatology and periodontal problems [7].

Hydrocortisone has more glucocorticoid effects than mineralocorticoid effects and has the least effect on leukocyte chemotaxis, and it is widely used in oral surgery [8]. Blackwell et al. [9] and Hong et al. [10] have shown that glucocorticoids will aid in the release of anti-phospholipase proteins, which inhibits the release of arachidonic acid and thus prevents the metabolism of prostaglandins and thromboxanes. However, limited research has directly compared the effectiveness of these interventions in terms of post-operative pain management and patient outcomes following mandibular molar extractions.

The study aimed to evaluate the efficacy of oxytetracycline hydrocortisone-soaked gauze in reducing post-operative pain and swelling compared to conventional surgical procedures without any packing in patients undergoing surgical extraction of the impacted mandibular third molars. The results of this study will help manage post-operative pain and swelling in cases of mandibular third molar surgical extractions and aid in achieving evidence-based treatment regimens. Identifying the most effective adjunctive therapy can improve patient comfort, reduce post-operative complications, and enhance overall treatment outcomes.

## Materials and methods

Study design and setting

This prospective study was conducted at Saveetha Dental College, Chennai - Tamil Nadu, in the Department of Oral and Maxillofacial Surgery. The study got approval from the Institutional Human Ethical Committee (IHEC) with the reference number - IHEC/SDC/OMFS-2204/23/161. The sample size was calculated using the G-power software with a confidence interval of 95%. Using simple randomization, fifty participants were included in the study, and they were divided into group A (gauze insertion group) in which after the surgical extraction of the impacted third molars, a gauze pack of 5*2 centimeters soaked with oxytetracycline hydrocortisone (10mg of hydrocortisone and 30mg of oxytetracycline) was inserted and closure done using the silk sutures. No gauze pack was placed in group B, and conventional closure was done. A single surgeon performed all the surgical procedures. Similar post-operative medications (Amoxicillin and Aceclofenac/Paracetamol combination) and instructions were given to patients in both groups.

Inclusion criteria

Patients in the age range of 20-40 years, irrespective of gender and requiring surgical removal of impacted lower third molar teeth with position A, class I mesioangular type, and who were categorized under American Society of Anesthesiology (ASA) class I was enrolled in the study.

Exclusion criteria

Patients who are medically compromised like diabetes and hypertension, smokers, alcoholics, pregnant females, anxious patients, patients with a history of allergy to anesthetics, tetracyclines, and hydrocortisone, presence of inflammation at the Surgical site, and the presence of prosthesis in the oral cavity were excluded from the study.

Surgical procedure

**Figure 1:**
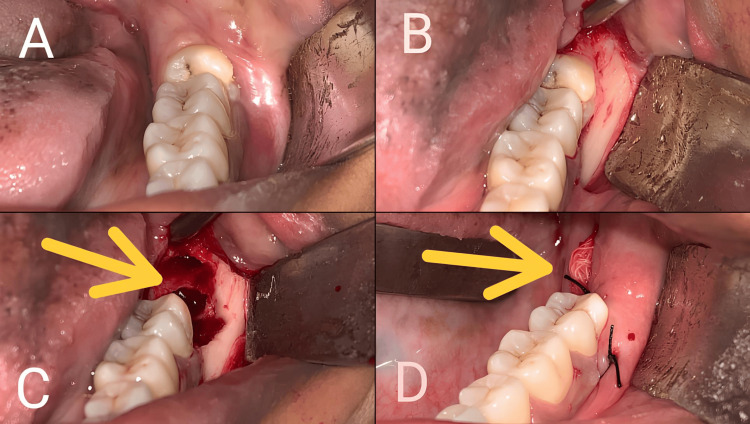
Surgical extraction of impacted lower third molar and placement of oxytetracycline hydrocortisone-soaked gauze in Group A A. Preoperative picture B. Mucoperiosteal flap elevation C. Socket after tooth extraction D. Oxytetracycline hydrocortisone-soaked gauze placed in the extraction socket in Group A

Under standard aseptic conditions, patient draping was done, and the patient was asked to gargle using the chlorhexidine mouthwash. With 2% lignocaine with the adrenaline of 1:80000, the inferior alveolar nerve block and the lingual and long buccal nerve block were given. Ward's incision was given, the full-thickness mucoperiosteal flap was elevated, buccal bone guttering was done using 702 bur, and the tooth was elevated and extracted. After tooth extraction, the socket was irrigated using normal saline. After achieving hemostasis in group A, a gauze pack of 5*2 cm soaked with oxytetracycline hydrocortisone, which contains 30 mg of oxytetracycline, and 10 mg of hydrocortisone was inserted in the socket, and closure was done using the silk suture (Figure 1). Whereas in group B patients, conventional closure was done without any pack insertion after achieving hemostasis. Post-operative instructions were given to the patients, who were instructed not to use any mouthwash. Patients were recalled on every alternative day. On post-operative day seven, intraoral irrigation was done using normal saline, and oxytetracycline hydrocortisone-soaked gauze was removed in group A. 

Outcome parameters

Clinical parameters that were assessed in the study are post-operative pain and swelling. Post-operative pain was assessed on days one, three, and five using the 10-point Visual analog scale. Post-operative swelling was assessed using a four-point measurement, including measurement from the corner of the mouth to the tragus of the ear and lateral canthus of the eye to soft tissue gonion on day three and day seven.

Statistical analysis

The normality between the two groups was assessed using the Shapiro-Wilk test. Data analysis was done using the SPSS software version 22.0 for Windows (IBM Corp., Armonk, NY). The data between the two groups was interpreted using an unpaired t-test with p<0.05 considered statistically significant.

## Results

Among 50 participants, 34 were males and 16 were females, with a mean age of 28 ± 4.2 years. Twenty-five participants were included in each group. In group A (N=25), gauze soaked with oxytetracycline hydrocortisone was used, whereas in group B (N=25), conventional closure was done.

Postoperative pain measurement

Post-operative pain assessment was done on post-operative day (POD) 1, POD 3, and POD 5 using the 10-point visual analog scale, and the results inferred are depicted in Table 1.

**Table 1:** Comparison of postoperative pain scores among the two study groups. Group A - Oxytetracycline hydrocortisone-soaked gauze insertion group; Group B - Conventional closure group. ** - Statistically significant.

Visual analog scale scores at different timelines	Groups	Number	Mean	Standard Deviation	p-value
Postoperative Day 1	Group A	25	4.14	0.82	0.001**
	Group B	25	5.2	1.19	
Postoperative Day 3	Group A	25	2.47	1.04	0.001**
	Group B	25	3.45	0.98	
Postoperative Day 5	Group A	25	1.19	0.86	0.001**
	Group B	25	2.18	0.81	

It was found that study participants of group A (oxytetracycline hydrocortisone) showed less post-operative pain compared to that of group B (conventional closure) on day 1, day 3, and day 5, and the results were statistically significant (p=0.001).

Measurement of post-operative swelling

A four-point facial measurement was done on POD 3 and POD 7 to evaluate the swelling. The data obtained is depicted in Table 2.

**Table 2:** Comparison of postoperative swelling values among the two study groups. Group A - Oxytetracycline hydrocortisone-soaked gauze group; Group B - Conventional closure. **  - Statistically significant; NS - not significant

Postoperative swelling values at different timelines	Groups	Number	Mean	Standard Deviation	p-value
Postoperative Day 3	Group A	25	9.15	0.347	0.001**
	Group B	25	10.83	0.382	
Postoperative Day 7	Group A	25	8.5	0.289	0.07
	Group B	25	8.45	0.301	NS

It was found that study participants of group A (oxytetracycline hydrocortisone) showed less post-operative swelling compared to that of group B (conventional closure) on day 3, and the results were statistically significant (p=0.001). In contrast, the difference in swelling between the two groups on POD 7 was not statistically significant (p=0.07).

## Discussion

In cases of surgical extraction, the post-operative complication rate varies between 2.6% and 30.9%, which includes bleeding, swelling (edema), persistent pain, trismus, and nerve injury [11]. Age, sex, impacted tooth level, surgical techniques, and operator skills are the factors that are recognized as potential risk factors for post-operative complications [12]. Even though these complications are considered treatable, the oral surgeon must be aware of abnormalities that may lead to prolonged complications [13]. Apart from these complications, dry socket, also called alveolar osteitis, is another cause that can lead to post-operative pain [14]. The incidence of dry sockets varies at different oral cavity sites, with 3% in general, and the incidence rate is more than 30% in the mandibular third molar region [15].

Various studies were done to prevent the occurrence of dry sockets in the mandibular molar region. Akota et al. found that placing the chlortetracycline-soaked gauze in the third molar socket significantly reduced pain and a 35% reduction in the dry socket formation [16]. Jr. Ragno et al. observed that when mouthwash containing 0.12% chlorhexidine was administered, there was a significant decrease in the incidence of dry socket and post-operative discomfort [17]. Coello Gomez et al. conducted a study in which chlorhexidine gel was used, and it was reported that there was a significant reduction in the visual analog scale score [18].

Apart from oxytetracycline, hydrocortisone has an anti-inflammatory role that aids in reducing swelling at the site of surgery. A study conducted by Baxendale et al. demonstrated that there was significant effectiveness in the reduction of post-operative swelling upon administration of 8 mg dexamethasone orally [19]. Beirne and Hollander, in their studies, found that administration of 125mg of methylprednisolone one hour before the procedure through the intravenous (IV) route has shown a significant reduction in swelling [20]. Similar results were observed by Holland upon administration of 40 mg dosage of IV methylprednisolone before surgery, in comparison to placebo results were statistically significant for inflammation (P=0.003) and pain (P<0.05) after 24 hours [21]. Compared to the studies mentioned above in our studies, we have delivered the drug at the local site of surgery, which enhances the treatment outcome.

Milles and Desjardins found that methylprednisolone effectively reduces post-operative inflammation compared to the placebo group (P=001) at 24 hours and 48 hours post-surgery [22]. Buyukkurt et al. also reported similar results [23]. Similarly, Skjelbred and Lokken found that an Intramuscular injection of 9 mg betamethasone after surgery has shown reduced pain (P<0.05) and inflammation (P< 0.05) [24]. Pedersen found that preoperative intramuscular injection of 4 mg dexamethasone over masseter muscle resulted in a 50% reduction in edema and a 30% reduction in pain [25].

Our study observed a statistically significant reduction in postoperative pain in the oxytetracycline hydrocortisone-gauze pack group on the first, third, and fifth postoperative days compared to the conventional closure group. Post-operative swelling was less in the oxytetracycline hydrocortisone-gauze pack group compared to the conventional closure group only on the third post-operative day. The difference in postoperative swelling between the two groups was not statistically significant on the seventh postoperative day.

Limitations of the study

The limitations of the study were it was conducted on a small population. In further studies, the sample size has to be increased. The study was also conducted at a single center; further studies must be conducted at multiple centers.

## Conclusions

Placement of the oxytetracycline hydrocortisone-soaked gauze in the extraction sockets aids in local drug delivery at the site of surgery. Oxytetracycline has antibiotic properties, which mainly reduce the microbial count at the site of surgery. On the other hand, hydrocortisone has anti-inflammatory properties; thus, it reduces post-operative swelling and pain. Apart from the local drug delivery action, placing the gauze in the extraction socket will prevent dead space formation and aid in granulation and healing. So, compared to conventional closure after surgical extraction of an impacted tooth, an oxytetracycline hydrocortisone pack can be recommended as it enhances the patient's quality of life.
